# The interactive role of methane beyond a reactant in crude oil upgrading

**DOI:** 10.1038/s42004-021-00590-3

**Published:** 2021-11-03

**Authors:** Hao Xu, Zhaofei Li, Yimeng Li, Hua Song

**Affiliations:** Green Catalysis Research Group, Department of Chemical and Petroleum Engineering, University of Calgary, 2500 University Drive, NW, Calgary, AB T2N 1N4 Canada

**Keywords:** Heterogeneous catalysis, Natural gas, Crude oil

## Abstract

Crude oil upgrading under methane has been reported to be an economically and environmentally promising process, while the advantageous effect of methane beyond a reactant is not fully explained. In this work, the catalytic performances, physicochemical properties and regenerability of used catalysts after crude oil upgrading under methane and nitrogen are investigated by *n*-butylbenzene model compound studies, catalyst characterizations and density functional theory calculations. Comparing to nitrogen, methane exhibits a protective effect on the charged catalyst despite the limited conversion, leading to better product quality and catalyst stability. This protective effect is attributed to the interaction between methane and catalytic active sites, which mainly occurs in the internal pores of the zeolitic catalyst support, resulting in unique coke distribution and inhibition of metal deposition. The interactive role of methane beyond a reactant, which is previously underestimated, is suggested to be critical for better performances of catalysts in relevant reaction processes.

## Abstract (web summary)

Crude oil upgrading under a methane environment can lead to improved oil quality as well as catalyst stability, but the role of methane in the reaction remains poorly understood. Here, the authors show that methane prevents coke formation inside the pores of the charged catalyst due to preferable adsorption.

## Introduction

With the depletion of fossil fuel resources, better utilization of raw natural materials such as crude oil attracts more and more global attention^1^. An upgrading process is generally necessary for the treatment of crude oils, especially heavy crudes, in which the properties of oils are greatly enhanced for downstream processing^2,3^. Hydrogen gas is mainly used as a hydrogen donor for the upgrading process to compensate for the low H:C ratio and remove heteroatoms present in the crudes^4,5^. Although satisfactory product quality can be obtained under a hydrogen atmosphere, hydrogen is not a naturally available resource and a methane reforming process is widely needed for its production^6^. However, this process often requires severe reaction conditions, and the consequent high cost and greenhouse gas emission lead to low profitability and environmental unfriendliness^7^. One promising solution is to directly use methane as an alternative hydrogen donor to replace hydrogen in oil upgrading processes. Methane, as the main component in natural gas, has been demonstrated to be with an abundant reserve and underestimated value^8–10^. The technical feasibility of methane-assisted oil upgrading processes has been well documented by our research group, in which methane leads to notably improved quality of oil products^11–17^. It is well studied that one common advantage of methane introduction is the participation of methane as a reactant in the upgrading process through a variety of pathways such as hydrogen transfer, alkylation and co-aromatization^14,18–20^. However, according to previous reports, the conversion of methane in oil upgrading processes remains at a relatively low level, especially when the reaction is performed under mild conditions^11,21,22^. Therefore, only limited incorporation of methane in final oil products is observed, which cannot fully explain the greatly improved oil quality^11,17,23^. Besides, the solvent effect of supercritical methane for coke inhibition has also been confirmed to be limited^23^. It seems that the presence of methane might have a more profound influence on the reaction process. Therefore, a more in-depth understanding of the role played by methane, which could provide more insights into its utilization, is of great significance.

In this work, the role of methane in the oil upgrading process beyond a reactant was investigated in several steps. A wide variety of techniques including thermalgravimetric analysis (TGA), derivative thermalgravimetric analysis (DTG), inductively coupled plasma-optical emission spectrometry (ICP-OES), total acid number (TAN) titration, simulated distillation analysis (SDA), micro-chromatography (micro-GC), gas chromatography–mass spectrometry (GC-MS), nitrogen adsorption–desorption, ammonia-temperature programmed desorption (NH_3_-TPD), high-angle annular dark-field scanning transmission electron microscopy (HAADF-STEM), and energy-dispersive X-ray spectroscopy (EDX) were used for product analysis and catalyst characterization. First, heavy oil upgrading experiments were carried out under both methane and nitrogen for several reaction cycles to get catalysts with different extents of deactivation. Then, *n*-butylbenzene was selected as a model compound to further evaluate and differentiate the catalytic performances of the spent catalyst collected under methane and nitrogen after different reaction cycles. *n*-butylbenzene was widely accepted as a model compound for the study of oil upgrading processes due to its unique structure^24,25^. The aromatic ring is representative of the presence of aromatics in crude oil, while the relatively long side chain is capable of producing light gaseous hydrocarbons, which are active and crucial for the evolution of oil molecules during both thermal and catalytic processes. Subsequently, the physical and chemical properties of the catalysts at their various life stages were characterized and compared, based on which the reasons for the different catalytic performances were explained. A simple regeneration process was also applied to the used catalysts and better regenerability of the catalyst previously used under methane was confirmed compared to that of the counterpart used under nitrogen. According to the results, the protective effect of methane for maintaining the catalyst activity was better interpreted and the conclusion was further supported by density functional theory (DFT) calculations. This work provides new perspectives into the role methane played in improving the quality of products and prolonging the lifetime of catalysts, which demonstrates the great potential of methane utilization beyond a reactant for various catalytic processes.

## Results and discussion

### Heavy oil upgrading reactions

First, heavy oil upgrading reactions over fresh MOU (methane-assisted oil upgrading) catalyst were repeated three times under both methane and nitrogen, and the results are shown in Table S1. High repeatability and acceptable mass balances are achieved under both environments, indicating the reliability of experimental operation and data analysis. The results are also used to evaluate the data uncertainty afterward. High liquid yield and limited coke yield can be observed, together with evidently decreased viscosity and density, preliminarily confirming that heavy oil has been successfully upgraded.

**Fig. 1:**
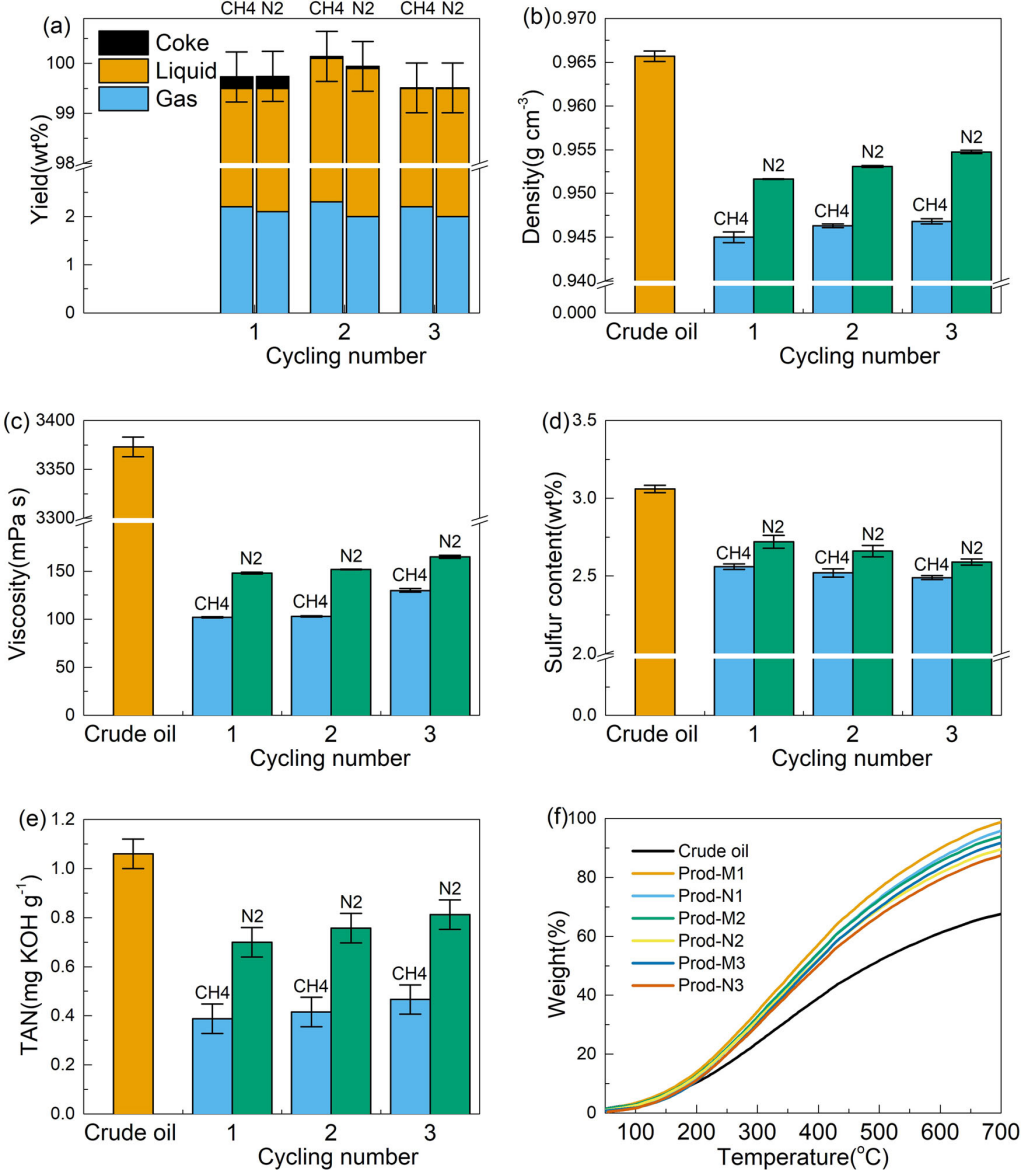
Properties of crude oil and upgrading products over catalysts with different reaction cycles under methane and nitrogen. **a** Gas, liquid, and coke yield, **b** density, **c** viscosity, **d** sulfur content, **e** TAN, **f** boiling curve derived from SDA. The error bars represent the standard deviation of the measurements.

Then, the MOU catalyst was used for 1–3 reaction cycles under both methane and nitrogen, and the properties of product oils are illustrated in Fig. 1. According to Fig. 1a, similar gas, liquid, and coke yields can be observed under methane and nitrogen. With increasing cycling numbers, gas and liquid yields remain constant. However, the coke yield decreases dramatically, leading to almost undetectable coke formation in the third cycle. This phenomenon can be due to the fact that, although coke tends to form over the fresh catalyst with abundant acid sites and well-established pore structures, the coking effect is remarkably inhibited over used catalyst due to acid site coverage and pore blockage. Figure 1b–e illustrates that the properties of oil products are unexceptionally better under methane comparing to nitrogen, in terms of density, viscosity, TAN and sulfur content. Moreover, according to the boiling curves in Fig. 1f, a noticeable lightening effect can be observed under both methane and nitrogen, evidenced by the shift of the patterns to the lower temperature from crude oil to products. For the same reaction cycle, this shift is more significant under methane than that under nitrogen, again suggesting that more light fractions can be produced in the presence of methane. These conclusions are also quantitatively supported by the reduced initial boiling points, increased recovery and lowered average molecular weight, particularly under methane, as shown in Table 1. The advantageous addition of methane in the oil upgrading process has been repeatedly confirmed in our previous studies^11,23,26^, which is usually explained by the reactions between methane and oil molecules^14,18–20^. However, the reported methane conversion is extremely limited (< 5%) in general^11,21,22^, suggesting that only a small portion of methane could be incorporated into final upgrading products, which cannot fully explain the tremendously improved product quality. The role of methane beyond a reactant in the oil upgrading process deserves extra investigation and will be discussed in the following sections.

**Table 1:** Typical SDA results for crude oil and upgrading products over catalysts with different reaction cycles under methane and nitrogen.

Property	Crude oil	Prod-M1	Prod-N1	Prod-M2	Prod-N2	Prod-M3	Prod-N3
Initial boiling point (°C)	54.4	34.1	40.9	33.9	35.3	34.3	37.6
Recovery at 700 °C (wt%)	68	99	96	94	89	92	87
Average molecular weight (g mol^−1^)	493.2	392.9	397.3	393.5	398.8	401.8	404.0

### Catalytic *n*-butylbenzene conversion

To elucidate the possible influence of methane on the catalyst performances, *n*-butylbenzene was used as an established model compound to better evaluate the used catalysts with different reaction cycles for heavy oil upgrading under methane and nitrogen. The overall analysis results are shown in Table S2. The mass balances of all entries are all met with acceptable uncertainty, indicating that sample collection and analysis methods are reasonable. Fresh MOU catalyst demonstrates high *n*-butylbenzene conversion, together with high gas yield, suggesting its high activity. The *n*-butylbenzene conversion is notably reduced after the catalyst is used for heavy oil upgrading, which keeps decreasing along with increasing reaction cycles. The lowest conversion is observed over silicon carbide (SiC), which is usually regarded as an unreactive solid to represent the results of pure thermal cracking. Comparing the results collected over spent catalysts, it is obvious that the *n*-butylbenzene conversion is significantly higher over catalyst used under methane than that used under nitrogen after the same reaction cycle. For example, the *n*-butylbenzene conversion in entry Cat-N3 is quite close to that in entry SiC, indicating the catalyst might have been fully deactivated after three consecutive runs under a nitrogen atmosphere. However, the conversion in entry Cat-M3 is even higher than that in Cat-N2, strongly demonstrating that the catalytic reaction process is better maintained in the presence of methane. Interestingly, despite the different *n*-butylbenzene conversions, all spent catalysts used in methane and nitrogen atmosphere with the same reaction cycle demonstrate similar coke yield for both oil upgrading and *n*-butylbenzene conversion reactions (Fig. 1a and Table S2), suggesting a similar extent of coking reactions. Given the fact that a comparable amount of coke is formed over all used catalysts with the same reaction cycle, the reason why catalysts under methane perform better deserves additional investigation.

The analysis of gas products for the conversion of *n*-butylbenzene is shown in Table S3. Higher methane conversion and yields of gas products (especially C_3_ and C_4_) are observed over the fresh catalyst, coinciding with the overall analysis results in Table S2. However, no significant difference is observed over the catalyst used under methane and nitrogen, in terms of methane conversion and the yields of other gas species. The results are also similar to those over SiC, suggesting that these gas products are mainly generated from the thermal cracking process.

**Fig. 2:**
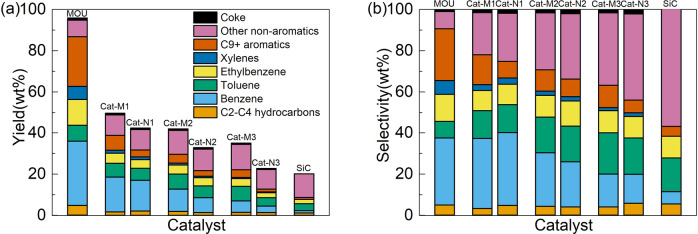
Product distribution of *n*-butylbenzene reactions over catalysts with different reaction cycles. **a** Yield, **b** selectivity. The unconverted *n*-butylbenzene has been excluded from the product.

The composition of liquid products was further analyzed and the product distribution is shown in Fig. 2. According to Fig. 2a, the fresh catalyst again presents distinctive performances, especially for the production of aromatics. Comparing the observed products over all fresh and used catalysts, the yields of aromatic species including benzene, toluene, ethylbenzene, xylenes (BTEX) and C_9+_ higher aromatics decreases along with the increasing number of reaction cycles. However, the yield of non-aromatic products (mainly paraffins and olefins) over all used catalysts remains unchanged or even enhanced, which is also close to that over SiC. Therefore, it can be clearly distinguished that aromatic products are mainly produced through the catalytic process while non-aromatic products are mostly derived from the pure thermal process. This conclusion has also been documented in previous work^26,27^. According to the mechanism studies, the thermal process mainly consists of the side chain breaking reactions, resulting in (alkyl)benzene and C_2_-C_4_ alkene or alkenylbenzene and C_1_-C_2_ alkane. The light hydrocarbons can further interact with each other through hydrogen transfer reactions. However, ring-opening reactions are not observed under the relatively mild reaction temperature (400 °C). With the presence of a catalyst, the cracking reactions are greatly intensified, leading to much higher *n*-butylbenzene conversion. Particularly, aromatization is widely accepted as the main function of zeolite catalysts^28–30^. The acid sites in the zeolite structure facilitate the protonation of aliphatic hydrocarbon chains derived from *n*-butylbenzene, triggering the dehydrogenation–oligomerization–aromatization process through hydrogen transfer among carbenium intermediates^31^. Moreover, the zeolite structure loaded with active metals leads to the activation of methane through asymmetric decomposition and generation of free methyl radicals, which can participate in various reactions including olefin saturation, co-aromatization, and methylation, further enhancing the yields of aromatics, particularly those with methyl substitution groups^18,32–36^. Therefore, the formation of aromatics can be used as an indicator to evaluate the activity of zeolite catalysts. It can be seen that the yields of aromatic products are significantly higher over the catalyst used under methane than those under nitrogen after the same reaction cycle, indicating the catalysts used under a methane atmosphere maintain higher activity. These conclusions become more evident when selectivity is considered (Fig. 2b): better catalyst activity leads to higher selectivity of aromatics; conversely, the selectivity of non-aromatic products increases along with the catalyst deactivation and is maximized in the thermal process over unreactive solid SiC. Since the methane conversion is limited and similar over all used catalysts as shown in Table S3, the different product distribution cannot be mainly attributed to the participation of methane in the reaction. Therefore, the obvious differences in product properties under methane and nitrogen suggest that the function of methane in upgrading processes is not limited to a reactant. The presence of methane might also profoundly influence the properties of the catalyst, leading to unique catalytic performances.

### Properties of used catalysts under methane and nitrogen

To investigate the reasons for different catalytic performances over used catalysts under methane and nitrogen, the physical and chemical properties of catalysts were further characterized. N_2_ adsorption isotherms in Fig. S1 clearly illustrate the different structural properties of catalysts, evidenced by the varied quantity of adsorbed nitrogen. Fresh catalyst demonstrates much higher adsorption capability, while the catalyst used under methane always behaves better than that under nitrogen with the same reaction cycle. The quantification results in Table 2 further confirm that the catalyst used under methane generally has a higher surface area and pore volume, especially micropore area and volume, than the counterpart under nitrogen, suggesting that the porous structure of the catalyst is better maintained in the presence of methane.

**Table 2:** Structural properties of fresh and used catalysts with different reaction cycles under methane and nitrogen.

Catalyst	BET surface area (m^2^ g^−1^)	Total pore volume (mL g^−1^)	Micropore surface area (m^2^ g^−1^)	Micropore volume (mL g^−1^)
MOU	179.8	0.205	82.7	0.043
Cat-M1	88.5	0.063	36.5	0.021
Cat-N1	69	0.058	16.3	0.005
Cat-M2	38.1	0.029	12.7	0.007
Cat-N2	36.5	0.025	7.1	0.005
Cat-M3	3.5	0.004	2.4	0.001
Cat-N3	0.4	0.001	0	0

NH_3_-TPD results in Fig. S2 also clearly differentiate the fresh and spent catalyst used for different cycles. High acidity is observed over the fresh catalyst, indicated by the large desorption peak. The peak intensity decreases rapidly over used catalysts, which can be due to the coverage of acid sites by the coking process. A small peak located at 430 °C can be observed over all used catalysts, which could be attributed to the desorption of high boiling point oil products since the catalyst has been used for heavy oil upgrading. The surface acidity of catalysts is further quantified and listed in Table 3. Higher acidity is maintained over catalyst used under methane than that under nitrogen with the same reaction cycle, indicating the capability of methane to maintain the active acid sites during the reaction process.

**Table 3:** Surface acidities of fresh and used catalysts with different reaction cycles under methane and nitrogen.

Catalyst	Surface acidity (μmol NH_3_ g^−1^)
MOU	311.1
Cat-M1	55.8
Cat-N1	49.8
Cat-M2	39.3
Cat-N2	38.5
Cat-M3	9.1
Cat-N3	2.8

Based on the results above, it is confirmed that significant catalyst deactivation can be observed after the oil upgrading process. Coke formation is widely accepted as one of the main reasons for catalyst deactivation, especially in the heavy oil upgrading process^37,38^. Given the fact that the amount of coke is similar over catalyst used under methane and nitrogen (Fig. 1a and Table S2), it can be hypothesized that the different locations of formed coke might be responsible for the different catalyst properties after using under methane and nitrogen.

**Fig. 3:**
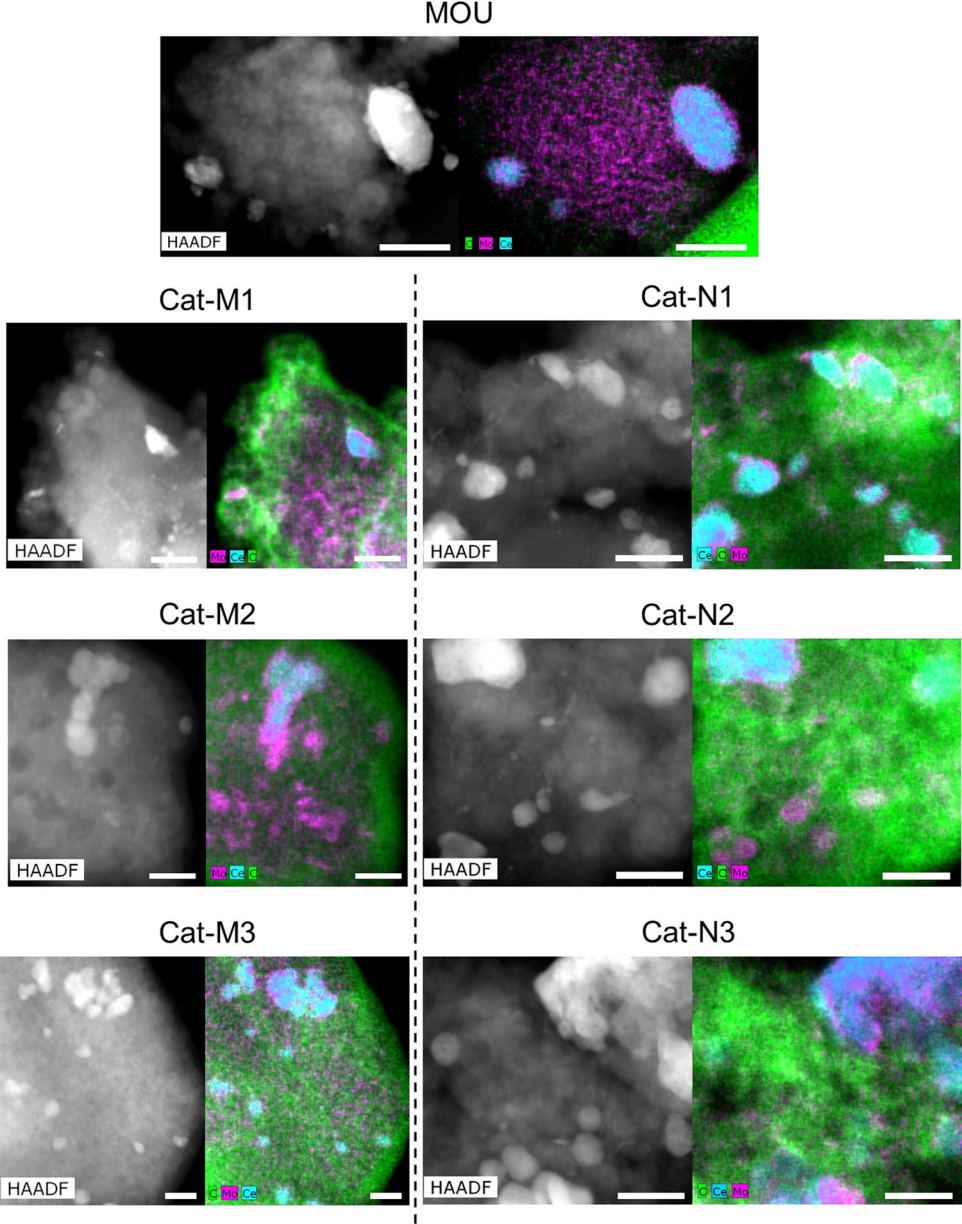
HAADF-STEM images and EDX mapping results of fresh and used catalysts with different reaction cycles under methane and nitrogen. Colors of elements: green for carbon, cyan for cerium, and magenta for molybdenum. All scale bars represent the length of 40 nm.

To verify the hypothesis, HAADF-STEM images and corresponding EDX mapping were acquired and the results are shown in Fig. 3. It can be seen that almost no carbon can be detected in fresh MOU catalyst (the carbon detected in the corner can be attributed to the lacey carbon grid used for sample loading), while conspicuous carbon signal can be observed over used catalysts. Comparing the used catalysts, it is found that the coke is mostly formed on the external surface of the catalyst after using under methane, indicated by the high carbon density at the periphery of the catalyst particles where the external surface is abundant. In contrast, the coke is formed all over the catalyst used under nitrogen, proven by the much more uniform carbon distribution. The different carbon distribution applies for all used catalysts under methane and nitrogen, regardless of the reaction cycles. Therefore, it can be concluded that the location of coke formation over the catalyst varies notably under methane and nitrogen. The preference of coke formation on the external surface of the catalyst effectively prevents the blockage of internal pore structure and coverage of active sites, explaining the different N_2_ adsorption/desorption and NH_3_-TPD results of used catalysts under methane and nitrogen. The unique coke distribution also implies that methane might preferably get adsorbed in the internal pore structure of the charged MOU catalyst, inhibiting the coke formation over there correspondingly.

The aforementioned conclusion is further supported by the DTG curves of the coke combustion process. According to Fig. S3, the coke formed over various catalysts conforms to a similar DTG pattern from 450 to 800 °C, which mainly derives from the polymerization of unsaturated species during the oil upgrading process^17^. Meanwhile, the peak shifts to a higher temperature along with increasing reaction cycle, which is also evidenced by the position of peak maximum as shown in Table 4. Interestingly, it is also indicated in Table 4 that the peak position of formed coke is constantly at a lower temperature over catalyst used under methane than that used under nitrogen with the same reaction cycle. Since peak position at lower temperature implies poorer thermal stability, it is anticipated that the coke formed under methane can be more easily removed through combustion, which is beneficial for catalyst regeneration. Since the coke on the external surface combusts much faster than that in the internal pore structure of the catalyst due to less mass and heat transfer resistances, the peak position at lower temperature also suggests the consistent conclusion that coke formation might preferably occur on the external surface of the catalyst in the presence of methane.

**Table 4:** Peak positions of formed coke in DTG curves over used catalysts with different reaction cycles under methane and nitrogen.

Catalyst	Peak position (°C)
Cat-M1	533
Cat-N1	546
Cat-M2	548
Cat-N2	561
Cat-M3	577
Cat-N3	592

**Fig. 4:**
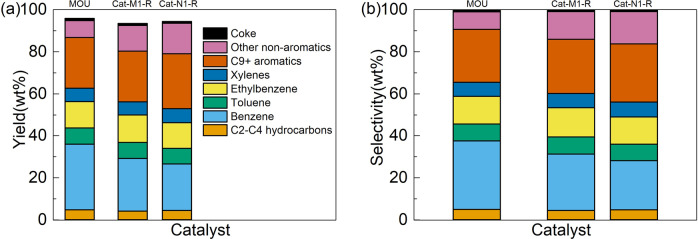
Product distribution of *n*-butylbenzene reactions over regenerated catalysts. **a** Yield, **b** selectivity. The unconverted *n*-butylbenzene has been excluded from the product, and the results over fresh catalyst are also presented for easier comparison.

The contents of several typical metal impurities deposited onto the catalysts collected at various life stages are also analyzed and listed in Table 5. These metal elements, including Ca, V, and Ni, are reported to be widely present in crude oils^39^, which could result in metal deposition over the used catalyst, leading to possible catalyst poisoning and deactivation. The impurities already present in fresh MOU catalyst might be introduced from the industrial catalyst fabrication process, while the metal deposition is evidenced by the increase of metal contents in used catalysts. Comparing the used catalysts, a general trend can be found that the metal contents are lower over the catalyst used under methane than that used under nitrogen with the same reaction cycle, suggesting that the presence of methane also partially inhibits the metal deposition over catalyst during the oil upgrading process. The deposition of the aforementioned metal impurities has been reported to exert adverse influences on catalytic processes. Ca, as an alkaline metal, is suggested to neutralize the acidic sites and form unreactive salts over the catalyst, leading to catalyst deactivation^40^. V is also reported to be strongly bound to active metals on the surface of the catalyst and facilitate the destruction of the zeolite framework, resulting in permanent catalyst deactivation^41–44^. Therefore, the reduced deposition of Ca and V could lessen the chemical deactivation of catalysts. This conclusion is supported by the higher activity of catalyst used under methane than that under nitrogen with the same reaction cycle in Fig. 2. Besides, metal depositions might also be closely correlated with coke formation. Although Ni is widely reported as an active component in various catalysts, it could also deposit homogeneously over the catalyst, causing irreversible catalyst deactivation, as well as create additional active sites for coking reactions^42–44^. Therefore, the prevention of Ni deposition could be advantageous for coke inhibition. It is also found that the presence of Ca facilitates the selective coking process inside the zeolite pore structure, which has been used as a technique for internal pore blockage^29,45,46^. Therefore, the less Ca content also explains the reduced coke formation in the internal pores as indicated by Fig. 4. Therefore, it can be concluded that the presence of methane mitigates the adverse effects caused by metal deposition, leading to better catalyst stability. Since the detrimental metal impurities contained in crude oil are inevitable, this effect is also highly beneficial for the adaptability and robustness of the involved catalytic oil upgrading processes.

**Table 5:** Contents of several typical metal impurities in fresh and used catalysts with different reaction cycles under methane and nitrogen.

Catalyst	Al (wt%)	Ca (ppm)	V (ppm)	Ni (ppm)
MOU	3.42 (3.42)	42	21	30
Cat-M1	3.42 (3.10)	97	113	87
Cat-N1	3.42 (3.08)	107	124	91
Cat-M2	3.42 (3.08)	109	121	151
Cat-N2	3.42 (3.06)	131	125	190
Cat-M3	3.42 (2.90)	116	129	198
Cat-N3	3.42 (2.89)	147	144	205

Al is used to calibrate the content of other elements on the basis of fresh catalyst and its content is therefore identical in all catalysts (the original content is given in parenthesis).

### Catalyst regeneration

Catalysts used under the methane and nitrogen environment were regenerated and used for *n*-butylbenzene conversion to evaluate the catalyst recyclability. The overall analysis, gas analysis, and liquid product distribution are given in Tables S4 and S5 and Fig. 4, respectively. The results in Table S4 indicate that similar *n*-butylbenzene conversion, as well as gas, liquid, and coke yield, can be achieved over regenerated catalyst regardless of the gas atmosphere of the previous run. The 93% *n*-butylbenzene conversion comes close to that over the fresh catalyst (96%), confirming that the catalyst can be successfully reused after a simple regeneration process. Table S5 also suggests that the composition of gas products is similar over regenerated catalysts. However, according to the liquid product distribution in Fig. 4, a clear difference between the catalyst previously used under methane and its nitrogen counterpart is observed. Higher yields of aromatic products, especially BTEX species, can be achieved over Cat-M1-R than those over Cat-N1-R, and the yields of non-aromatic products are lower correspondingly. As discussed above, the better activity of Cat-M1-R than Cat-N1-R is confirmed. This conclusion becomes more apparent after the fresh and regenerated catalysts are compared: the catalytic performance of Cat-M1-R is closer to the fresh catalyst MOU comparing with Cat-N1-R, again indicating the activity of the catalyst previously used under methane can be better recovered. Therefore, it can be concluded that the presence of methane also improves the regenerability of the catalyst for long-term usage. This improvement can be attributed to easier coke combustion and less severe metal deposition as shown above, which considerably enhances the potential of the technique for real industrial practices where catalyst regeneration is crucial.

### DFT calculation results

**Fig. 5:**
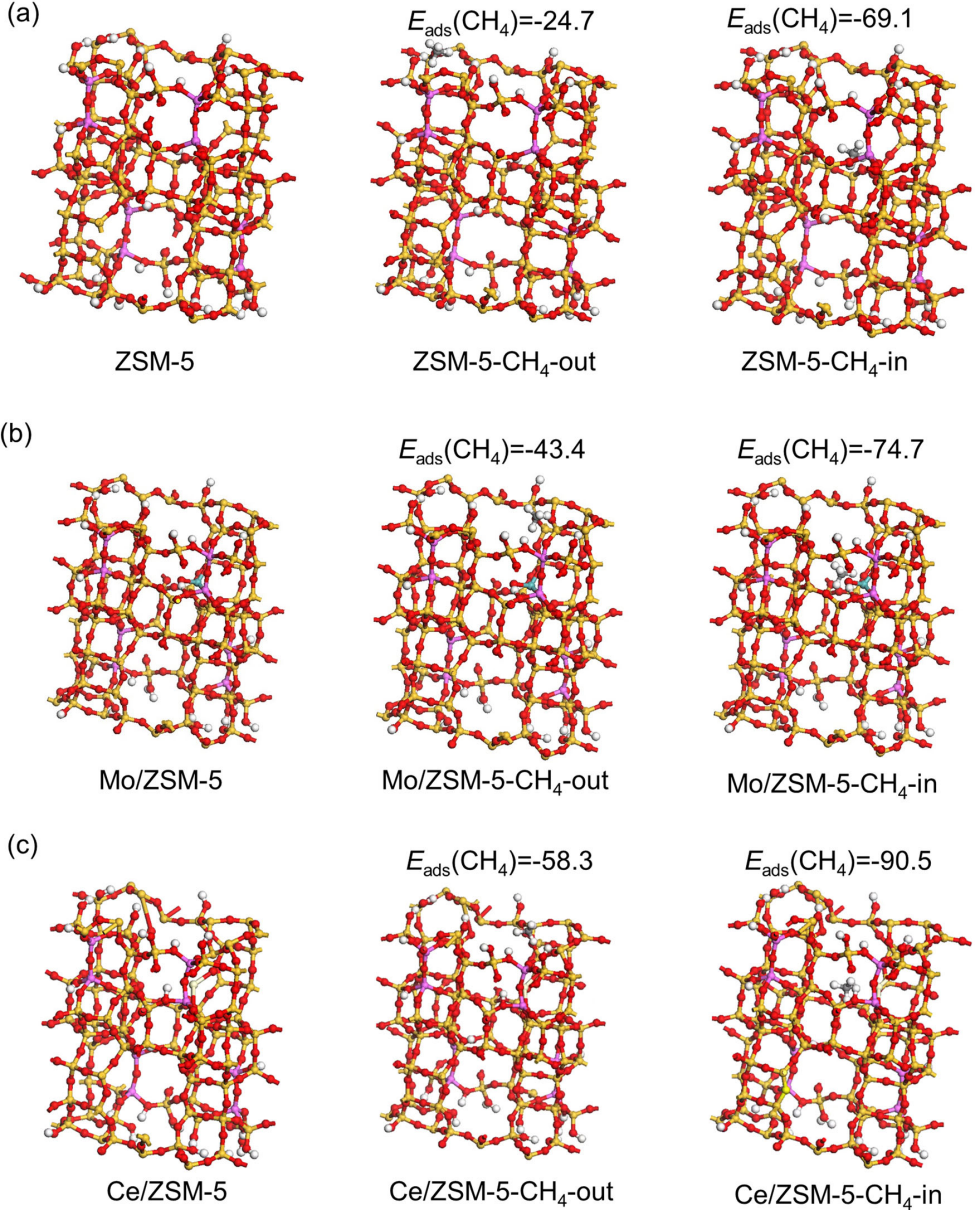
Optimized structures of catalysts and methane adsorption on the external surface and in the internal pore structure of the zeolitic catalysts through DFT calculation. **a** ZSM-5 catalyst, **b** Mo/ZSM-5, **c** Ce/ZSM-5. Energy unit: kJ mol^−1^.

DFT calculation was further performed to provide more clues for the selective adsorption of methane over the charged MOU catalyst. The adsorption energies of methane on the external surface and in the internal pore structure of ZSM-5 catalyst without and with the active metal site were obtained, respectively, after the geometry structures were optimized, as shown in Fig. 5. According to Fig. 5a, the adsorption energy of methane in the internal pore structure of zeolite catalyst is lower than that on the external surface, suggesting that the adsorption of methane inside the zeolite is favorable. Figure 5b, c indicates that the addition of the active metal Mo or Ce further enhances the adsorption energy (absolute value), leading to stronger methane adsorption. The enhanced methane adsorption often leads to better catalytic performances, which has been documented in the previous reports^16,26,47,48^. Meanwhile, the preferred methane adsorption in the internal pore structure remains unchanged regardless of the presence of active metal species. These DFT results are highly consistent with experimental observations, further validating the selective adsorption of methane, which is essential for relevant catalytic processes. Besides, the similar calculation was also performed using N_2_ as the adsorbate, and the results are shown in Fig. S4. Comparing Fig. 5 and Fig. S4, the adsorption of nitrogen is much weaker than that of methane, both on the external surface (−16.0 kJ mol^−1^ for nitrogen and −24.7 kJ mol^−1^ for methane) and in the internal pore structure of the catalyst (−17.5 kJ mol^−1^ for nitrogen and −69.1 kJ mol^−1^ for methane). Besides, the adsorption energy difference of nitrogen (1.5 kJ mol^−1^) outside and inside the pore is also much smaller than that of methane (44.4 kJ mol^−1^). Therefore, it can be concluded that nitrogen has less interaction with the catalyst than methane, and the preference of adsorption inside the pore structure is also less significant, coinciding with all the results discussed above.

The corresponding calculations were also carried out using *n*-butylbenzene as the adsorbate and the results are presented in Fig. S5. Similar conclusions can be drawn that *n*-butylbenzene also prefers to be adsorbed in the internal pore structure of zeolite catalyst, and the addition of active metals further facilitates the adsorption. The Ortmann–Bechstedt–Schmidt dispersion corrections were also considered for the calculation of *n*-butylbenzene adsorption over the catalyst, and the results are shown in Table S6. According to Table S6, slightly different adsorption energies are obtained after dispersion correction, while the conclusions derived from Fig. S5 still remain valid. It should be noted that the adsorption energies of *n*-butylbenzene are much higher (absolute value) than those of methane, indicating the adsorption of *n*-butylbenzene can be much more favorable than methane under similar conditions. This is consistent with the observation that the conversion of *n*-butylbenzene (96%) is much higher than that of methane (2.1%) over the fresh catalyst. However, the concentration of *n*-butylbenzene (0.07 mol L^−1^) is much lower than that of methane (1.9 mol L^−1^) under the reaction conditions. Besides, from a kinetic point of view, *n*-butylbenzene is a relatively large molecule with steric effect and mass transfer limitations over the zeolite catalyst, and thus not each *n*-butylbenzene molecule can effectively interact with the catalyst. In contrast, these limitations for methane are much less significant. Therefore, the interaction between methane and the catalyst still plays an important role despite the differences in adsorption energies. These results further explain the observation that better activity can be observed over catalyst used under methane, since the active sites protected by methane are also capable of *n*-butylbenzene conversion. The adsorption preference consistency of methane and *n*-butylbenzene further suggests that selective methane adsorption is highly useful and effective for improving the catalytic process by facilitating the reactions of other relevant reactants.

### Role of methane beyond a reactant: new perspectives

According to the aforementioned results, the effect of maintaining the activity of the catalyst in the presence of methane can be better interpreted. It has been previously reported that methane can interact with the active metals and acidic sites in zeolite-based catalysts through at least two possible pathways, which can be called the carbenium (CH_3_^+^) pathway and alkyl (CH_3_^−^) pathway^48–50^. Comparing to the unreactive nitrogen environment, the interaction between methane and catalyst occupies the active sites over the catalyst. This interaction can be beneficial for inhibiting detrimental metal deposition onto the active sites, as well as reducing the excessive adsorption of other reactants (especially unsaturated species) which finally leads to coking deactivation. This interaction preferably occurs in the internal pore structure of zeolite catalyst, where abundant active sites exist. This selective adsorption of methane is consistent with previous reports^19,21^, which is also supported by DFT calculations. Moreover, the activated methane molecules could in turn participate in the reaction process as reported previously^18,20^. However, although methane participation has been well studied, the role of methane in protecting the active sites during the reaction is often overlooked, which could result in a significant underestimation of the positive effect after methane introduction. It can be anticipated that the role of methane beyond a reactant opens up new opportunities for its wider utilization in various catalytic processes.

## Conclusions

The role of methane in the heavy oil upgrading process is understood from new perspectives by collecting used catalysts after 1–3 reaction cycles, subsequent *n*-butylbenzene model compound study, and catalyst characterizations. It is found that, comparing to unreactive nitrogen, the presence of methane leads to improved properties of oil products, agreeing with previous studies. The *n*-butylbenzene conversion reactions suggest that, besides the participation as a reactant in the reaction network, methane also acts as a protective agent to effectively maintain the activity of catalyst. This protective effect is further attributed to the maintenance of the physical and chemical properties of the charged catalyst as well as the reduced deposition of metal impurities from oil samples, which slow down the deactivation process. Since the coking effect is one of the most important reasons for catalyst deactivation, the distribution of coke over used catalysts is also observed. Comparing to the universal coke formation all over the catalyst after using under nitrogen, the coke tends to be formed mainly on the external surface of the catalyst in the presence of methane. The unique properties of catalyst used under methane can be due to the active interaction between methane and catalyst during the reaction, which preferably occurs in the internal pore structure of the catalyst. The preferred catalyst recyclability after using under methane is also confirmed by the experiments using regenerated catalysts. DFT calculations also suggest that the adsorption of methane and *n*-butylbenzene preferably occurs in the internal pore structure of the charged catalyst, coinciding with experimental observations. This work provides new insights into methane-assisted catalytic processes, in which the role of methane is beyond a reactant, and the favorable influence of methane on improving the product quality and maintaining the activity of catalysts can be of great significance in a wide variety of catalytic reaction processes.

## Methods

### Catalyst preparation

NH_3_-type ZSM-5 zeolite support with the SiO_2_ to Al_2_O_3_ ratio of 23:1 purchased from Zeolyst, USA was converted to H-type through calcination at 550 °C in static air for 5 h. Mo is reported to facilitate the asymmetrical decomposition of methane through the carbenium (CH_3_^+^) pathway^48^. Ce is reported to provide oxygen vacancies and inhibit coke formation^51^. These metals were thus selected and optimized as the active components for the catalyst. Mo and Ce derived from the precursor salts ammonium heptamolybdate tetrahydrate and cerium nitrate hexahydrate were loaded onto the support through impregnation, and the catalyst was then calcined at 550 °C for 2 h. The nominal loading of Mo and Ce were 10 wt% (weight percentage) and 5 wt%, respectively. After the calcination process, the Mo and Ce active components are mainly in oxide forms (i.e., MoO_3_ and CeO_2_), which has been confirmed in previous studies and are thus used in DFT calculations^47,48,51^. The prepared MoCe/ZSM-5 catalyst was designed and optimized in this study for methane-assisted oil upgrading and thus labeled as MOU.

### Reaction process

A 300 mL batch reactor (Parr Instrument, USA) was used for heavy oil upgrading processes. A typical heavy crude oil with a viscosity of 3373 cP and a density of 0.9657 g cm^−3^ at 15.6 °C was chosen as the feedstock. Additional physical and chemical properties of the heavy crude are listed in Table S7. In all, 0.70 g MOU catalyst and 70.00 g heavy oil were used for each run. The catalyst and heavy oil were directly mixed together in the reactor without using a catalyst basket. For experiments under a methane atmosphere, a standard gas with the composition of 90 mol% CH_4_ and 10 mol% N_2_ (Air Liquide) was used as the feed gas. The contained nitrogen gas in methane was used as an internal standard for the accurate calculation of methane conversion. In contrast, pure nitrogen (99.999%, Air Liquide) was used as an unreactive gas in control experiments. Before the reaction, the reactor was purged three times and pressurized to 3 MPa using the corresponding feed gas. To exclude the possibility of gas leakage, the weight and pressure of the reactor were also confirmed to be stable. The reactor was then heated up in a mantle equipped with a temperature controller and the stirring by a magnetic stirrer was kept at 120 rpm (revolutions per minute). The typical profiles of temperature and pressure during the reaction are shown in Fig. S6. It can be seen that it took around 30 min to reach the temperature setpoint 400 °C and the stable pressure under reaction conditions was around 6 MPa. The reaction lasted 1 h in the stable stage and an air flow was used to cool down the reactor afterward.

To study the catalyst deactivation process, catalysts were used with different cycling numbers. The used MOU catalyst collected after one heavy oil upgrading reaction under a methane environment was denoted as Cat-M1. The Cat-M1 catalyst was washed by toluene and dried at 90 °C overnight and then used again for heavy oil upgrading reaction under a methane environment. The resultant catalyst was denoted as Cat-M2. Similarly, the catalyst collected after another heavy oil upgrading reaction using Cat-M2 was denoted as Cat-M3. Control experiments under a nitrogen environment were also carried out, and the corresponding used catalysts were labeled as Cat-N1, Cat-N2, and Cat-N3, respectively. The liquid oil products were labeled as Prod-M1, Prod-M2, Prod-M3, Prod-N1, Prod-N2, and Prod-N3, respectively, to differentiate from catalysts.

The catalytic performances over the abovementioned used catalysts were further evaluated by a model compound study using *n*-butylbenzene as the reactant. A 100 mL batch reactor (Parr Instrument, USA) was used for catalytic *n*-butylbenzene conversion. In all, 0.20 g catalyst and 1.00 g *n*-butylbenzene (> 99%, Sigma-Aldrich) were used for each run. A standard gas with the composition of 90 mol% CH_4_ and 10 mol% N_2_ (Air Liquide) was used as the feed gas. The contained nitrogen gas in methane was used as an internal standard for the accurate calculation of methane conversion. Before the reaction, the reactor was purged three times and pressurized to 3 MPa. To exclude the possibility of gas leakage, the weight and pressure of the reactor were also confirmed to be stable. The reactor was then heated up in a mantle equipped with a temperature controller. For the *n*-butylbenzene reactions, the typical profiles of temperature and pressure during the reaction are shown in Fig. S7. It can be seen that it took around 20 min to reach the temperature setpoint 400 °C and the stable pressure under reaction conditions was around 6 MPa. The reaction lasted 1 h in the stable stage and an air flow was used to cool down the reactor afterward. Fresh MOU catalyst was also evaluated for comparison. Moreover, a reaction over unreactive, non-porous solid SiC was also performed to simulate the results of pure thermal reaction. The experiments were labeled according to the name of the charged catalyst, namely MOU, Cat-M1, Cat-M2, Cat-M3, Cat-N1, Cat-N2, Cat-N3, and SiC.

Moreover, a simple regeneration process was also applied to catalysts used under methane and nitrogen environment. Typically, 0.50 g used catalyst was set in a muffle furnace under air atmosphere, heated up to 550 °C with a temperature ramping rate of 10 °C min^−1^ and held for 12 h. The regenerated catalysts from Cat-M1 and Cat-N1 were labeled as Cat-M1-R and Cat-N1-R, respectively.

### Product analyses

For heavy oil upgrading reactions, the reactor was degassed after the reaction, and the mass loss was recorded for gas yield calculation. Then the reactor was opened, and the product oil and used catalyst were collected and separated through centrifugation. The mass of the oil product was recorded for liquid yield calculation.

The qualities of oil samples were analyzed according to the following methods. The density was measured by a densimeter (Anton Paar DMA 4500M). The viscosity was measured by a viscometer (Fungilab). The TAN was determined by a volumetric titrator (Metrohm 848 Titrino Plus). Typically, 5.00 g sample was dissolved in 100 mL standard titration solution (75 vol% xylenes and 25 vol% isopropanol) and 0.1 mol L^−1^ KOH solution was added gradually until the sudden change of voltage measured by an electrode was observed. The TAN value was then calculated according to the consumed volume of the KOH solution. The sulfur content was measured by ICP-OES (iCAP 7000 SERIES, thermal scientific) after the sample was fully digested. Typically, 0.20 g sample was added in a Teflon liner together with 6 mL concentrated nitric acid, 2 mL concentrated hydrochloric acid, and 2 mL 30% hydrogen peroxide. Then the liner was sealed and put on a rotor designed for a microwave digestor (Anton Parr Multiwave 7000). The digestion was carried out at 250 °C for 1 h. SDA was realized through a GC (Agilent 8890) with a flame ionization detector. A 30 m HP5 column was used and helium was the carrier gas. Typically, 0.10 g sample was dissolved in 1.00 g carbon disulfide and sealed in a vial designed for sample injection. The column temperature was programmed to be ramped from −20 °C (liquid nitrogen cooling) to 450 °C at a rate of 10 °C min^−1^. The data were processed by the SimDis Expert software.

The used catalyst was further rinsed by toluene (> 99.5, ACS Reagent) several times to remove soluble products. Then, the coke amount was determined by TGA.

For reactions using *n*-butylbenzene as the model compound, the product gas was introduced into a micro-GC (Agilent 490) with a thermal conductivity detector for compositional analysis. The micro-GC was equipped with four channels for analyzing various gas products detailed as follows: Channel 1, 10 m molecular sieve 5A column for the analysis of hydrogen, oxygen, nitrogen, methane, and carbon monoxide with the working temperature of 80 °C and pressure of 200.0 kPa; Channel 2, 10 m PPU column for the analysis of carbon dioxide, ethane, and ethylene with the working temperature of 100 °C and pressure of 175.0 kPa; Channel 3, 10 m alumina column for the analysis of hydrocarbons with 4–6 carbons with the working temperature of 80 °C and pressure of 180.0 kPa; Channel 4, 8 m CP-Sil 5CB column for the analysis of propane and propylene with the working temperature of 100 °C and pressure of 85.0 kPa. The carrier gas was argon and the time for each analysis was 2.5 min. After degassing, the reactor was weighed and opened. Then liquid and solid products were first collected together, then separated by filtration through medium-rate qualitative filter paper. Next, 0.10 g liquid product was diluted in 0.90 g acetone (> 99.5%, Sigma-Aldrich) and analyzed by GC-MS (Perkin Elmer) to get the compositional information. An HP-PONA column from Agilent was used for sample separation and the peak designation was realized by matching the mass spectra with the standard substances in the National Institute of Standards and Technology database and choosing the best hit. The concentration of each species was quantified after the chromatogram was pre-calibrated by standard substances. The collected solid was rinsed with acetone several times and TGA was further performed to determine the coke yield.

Based on the data above, several typical values for evaluating the catalytic performances were calculated by the following equations:

$${{{{{{{\mathrm{Overall}}}}}}}}\;{{{{{{{\mathrm{mass}}}}}}}}\;{{{{{{{\mathrm{balance}}}}}}}} = \frac{{{{{{{{{\mathrm{mass}}}}}}}}\;{{{{{{{\mathrm{of}}}}}}\;({{{{{\mathrm{gas}}}}}}}} + {{{{{{{\mathrm{liquid}}}}}}}} + {{{{{{{\mathrm{solid}}}}}})\;{{{{{\mathrm{after}}}}}}}}\;{{{{{{{\mathrm{reaction}}}}}}}}}}{{{{{{{{{\mathrm{mass}}}}}}}}\;{{{{{{{\mathrm{of}}}}}}\;({{{{{\mathrm{gas}}}}}}}} + {{{{{{{\mathrm{liquid}}}}}}}} + {{{{{{{\mathrm{solid}}}}}})\;{{{{{\mathrm{before}}}}}}}}\;{{{{{{{\mathrm{reaction}}}}}}}}}} \times 100\%$$

$${{{{{{{\mathrm{Gas}}}}}}}}\,{{{{{{{\mathrm{yield}}}}}}}} = \frac{{{{{{{{{\mathrm{mass}}}}}}}}\,{{{{{{{\mathrm{of}}}}}}}}\,{{{{{{{\mathrm{produced}}}}}}}}\,{{{{{{{\mathrm{gas}}}}}}}}}}{{{{{{{{{\mathrm{mass}}}}}}}}\,{{{{{{{\mathrm{of}}}}}}}}\,{{{{{{{\mathrm{fed}}}}}}}}\,{{{{{{{\mathrm{liquid}}}}}}}}\,{{{{{{{\mathrm{feedstock}}}}}}}}}} \times 100\%$$

$${{{{{{{\mathrm{Liquid}}}}}}}}\,{{{{{{{\mathrm{yield}}}}}}}} = \frac{{{{{{{{{\mathrm{mass}}}}}}}}\,{{{{{{{\mathrm{of}}}}}}}}\,{{{{{{{\mathrm{liquid}}}}}}}}\,{{{{{{{\mathrm{product}}}}}}}}}}{{{{{{{{{\mathrm{mass}}}}}}}}\,{{{{{{{\mathrm{of}}}}}}}}\,{{{{{{{\mathrm{fed}}}}}}}}\,{{{{{{{\mathrm{liquid}}}}}}}}\,{{{{{{{\mathrm{feedstock}}}}}}}}}} \times 100\%$$

$${{{{{{{\mathrm{Coke}}}}}}}}\,{{{{{{{\mathrm{yield}}}}}}}} = \frac{{{{{{{{{\mathrm{mass}}}}}}}}\,{{{{{{{\mathrm{of}}}}}}}}\,{{{{{{{\mathrm{generated}}}}}}}}\,{{{{{{{\mathrm{coke}}}}}}}}}}{{{{{{{{{\mathrm{mass}}}}}}}}\,{{{{{{{\mathrm{of}}}}}}}}\,{{{{{{{\mathrm{fed}}}}}}}}\,{{{{{{{\mathrm{liquid}}}}}}}}\,{{{{{{{\mathrm{feedstock}}}}}}}}}} \times 100\%$$

$${{{{{{{\mathrm{Methane}}}}}}}}\,{{{{{{{\mathrm{conversion}}}}}}}} = \left( {{{{{{{{\mathrm{1 - }}}}}}}}\frac{{{{{{{{{\mathrm{mole}}}}}}}}\,{{{{{{{\mathrm{of}}}}}}}}\,{{{{{{{\mathrm{remaining}}}}}}}}\,{{{{{{{\mathrm{methane}}}}}}}}\,{{{{{{{\mathrm{after}}}}}}}}\,{{{{{{{\mathrm{reaction}}}}}}}}}}{{{{{{{{{\mathrm{mole}}}}}}}}\,{{{{{{{\mathrm{of}}}}}}}}\,{{{{{{{\mathrm{fed}}}}}}}}\,{{{{{{{\mathrm{methane}}}}}}}}}}} \right) \times 100\%$$

For *n*-butylbenzene reactions:

$${{{{{{{\mathrm{Butylbenzene}}}}}}}}\,{{{{{{{\mathrm{conversion}}}}}}}} = \left( {{{{{{{{\mathrm{1 - }}}}}}}}\frac{{{{{{{{{\mathrm{mole}}}}}}}}\,{{{{{{{\mathrm{of}}}}}}}}\,{{{{{{{\mathrm{remaining}}}}}}}}\,{{{{{{{\mathrm{butylbenzene}}}}}}}}\,{{{{{{{\mathrm{after}}}}}}}}\,{{{{{{{\mathrm{reaction}}}}}}}}}}{{{{{{{{{\mathrm{mole}}}}}}}}\,{{{{{{{\mathrm{of}}}}}}}}\,{{{{{{{\mathrm{fed}}}}}}}}\,{{{{{{{\mathrm{butylbenzene}}}}}}}}}}} \right) \times 100\%$$

$${{{{{{{\mathrm{Yield}}}}}}}}\,{{{{{{{\mathrm{of}}}}}}}}\,{{{{{{{\mathrm{product}}}}}}}}\,{{{{{{{\mathrm{P}}}}}}}} = \frac{{{{{{{{{\mathrm{mass}}}}}}}}\,{{{{{{{\mathrm{of}}}}}}}}\,{{{{{{{\mathrm{P}}}}}}}}}}{{{{{{{{{\mathrm{mass}}}}}}}}\,{{{{{{{\mathrm{of}}}}}}}}\,{{{{{{{\mathrm{fed}}}}}}}}\,{{{{{{{\mathrm{butylbenzene}}}}}}}}}} \times 100\%$$

$${{{{{{{\mathrm{Selectivity}}}}}}}}\,{{{{{{{\mathrm{of}}}}}}}}\,{{{{{{{\mathrm{product}}}}}}}}\,{{{{{{{\mathrm{P = }}}}}}}}\frac{{{{{{{{{\mathrm{mass}}}}}}}}\,{{{{{{{\mathrm{of}}}}}}}}\,{{{{{{{\mathrm{P}}}}}}}}}}{{{{{{{{{\mathrm{mass}}}}}}}}\,{{{{{{{\mathrm{of}}}}}}}}\,{{{{{{{\mathrm{converted}}}}}}}}\,{{{{{{{\mathrm{butylbenzene}}}}}}}}}} \times 100\%$$

### Catalyst characterizations

TGA was carried out for the quantification of coke content. Typically, 50 mg sample was heated up from 50 to 800 °C at a temperature ramping rate of 20 °C min^−1^ under 30 mL min^−1^ air flow and the weight change was recorded by a PerkinElmer STA 6000 thermal analyzer. DTG curves were also acquired to analyze the thermal stability of coke over different catalysts.

N_2_ adsorption–desorption was used to evaluate the structural properties of catalyst samples. Typically, 0.2 g sample was first degassed at 350 °C for 4 h and then analyzed in liquid nitrogen on ASAP 2020 plus (Micromeritics). The Brunauer–Emmett–Telle method was used to determine the total surface area and total pore volume was acquired at the relative pressure of 0.995. The *t*-plot method was used for micropore area and volume calculations.

NH_3_-TPD was used to determine the surface acidity of catalysts. Typically, 0.2 g sample was loaded into a U tube and set on a Finesorb-3010 automated chemisorption analyzer. The sample was first degassed at 350 °C with a 25 mL min^−1^ argon flow for 1 h. Then ammonia adsorption was performed by feeding 25 mL min^−1^ 10% NH_3_/Ar at 100 °C for 1 hour. Next, the gas was switched to argon again to remove the physically adsorbed ammonia for 30 min. Finally, the temperature was ramped from 100 to 800 °C at a rate of 10 °C min^−1^ and the desorption signals were monitored by a thermal conductivity detector.

STEM was performed on an FEI Tecnai Osiris S/TEM system equipped with Bruker SuperX EDX detectors. The operation voltage was 200 keV. The samples were dispersed in isopropanol (99.9% Sigma Aldrich) with the assistance of sonication and then cast dropwise on a 200 mesh Cu lacey formvar/carbon Ted Pella grid.

To confirm the extent of metal deposition, the contents of several commonly seen metal impurities from oil samples in the catalysts were also quantified by ICP-OES after the catalysts were fully digested. Typically, 0.20 g catalyst sample was added in a Teflon liner together with 6 mL concentrated nitric acid and 2 mL 50% hydrofluoric acid. Then the liner was sealed and put on a rotor designed for a microwave digestor (Anton Parr Multiwave 7000). The digestion was carried out at 250 °C for 1 h. All metal contents in used catalysts were calibrated and expressed on the basis of fresh catalyst assuming the content of Al was identical before and after the reaction, based on the method reported in a previous work^52^. To confirm the elemental balances, the contents of these metal impurities in crude oil and liquid products are also given in Table S8. It can be seen that no significant decreases of metal contents in the product oils are observed. This can be due to the low catalyst to feed ratio (1:100) and the relatively small amount of metal deposition over the catalyst so that the decreased contents in the products were within the experimental error (~5 ppm).

### DFT calculations

The DFT calculation was carried out using the DMol3 module in Materials Studio software (Accelrys Inc.)^53,54^, and the calculation process was similar to a previous publication^45^. The B3LYP hybrid functional and double numeric quality basis set were adopted to provide reasonable accuracy with acceptable computational expenses for zeolite catalysts, as reported in previous studies^55–57^. The tolerances were set to be 2 × 10^−5^ Ha for energy, 4 × 10^−3^ Ha Å^−1^ for gradient and 5 × 10^−3^ Å for displacement, respectively.

To simulate the zeolite support in this study, an aluminum-containing HZSM-5 zeolite model was selected based on previous reports^58–60^. The unit cell originally consisted of 96 Si atoms and 192 O atoms, and the lattice parameters were *a* = 20.090 Å, *b* = 19.378 Å, and *c* = 13.142 Å, respectively. Two Si atoms at the T-12 sites, where a high probability of replacement was reported, were replaced by Al in order to satisfy the silica to alumina ratio. To better differentiate the adsorption of methane outside and inside the pore structure, the unit cell was prolonged in the *b* direction of the triclinic crystal structure with a 20 Å vacuum layer. This direction was parallel with the straight channels of the zeolite structure, which was confirmed to be the most favorable for the diffusion of free molecules^61^. All terminal O atoms were saturated by H atoms.

The structure of zeolite was first optimized, followed by the addition of active Mo and Ce species in oxide form. The metal oxides were located in the straight channel of the zeolite structure next to the aforementioned acid sites. Then the adsorbate molecule (methane, nitrogen or *n*-butylbenzene) was added outside and inside the zeolitic structure with high proximity to the acid site as the initial geometrical guess, respectively. This initial geometrical input was confirmed to provide the final structure with the lowest energy after several other conformations (shown in Figs. S8 and S9) were attempted. After the geometry optimization, the adsorption energy was calculated by the following equation:

$$E_{{{{{{{{\mathrm{ads}}}}}}}}} = E_{{{{{{{{\mathrm{A}}}}}}\mbox{-}{{{{{\rm{C}}}}}}}}} - E_{{{{{{{\mathrm{A}}}}}}}} - E_{{{{{{{\mathrm{C}}}}}}}}$$

Where *E*_ads_ is the adsorption energy, *E*_A-C_ is the energy of the adsorbed adsorbate together with the catalyst structure, *E*_A_ is the energy of the free adsorbate molecule, and *E*_C_ is the energy of the catalyst structure in its original form.

## Supplementary information

Supplementary Information

## Data Availability

The datasets generated during and/or analyzed during the current study are available from the corresponding author on reasonable request.
