# Supplementary material for: The interactive role of methane beyond a reactant in crude oil upgrading
Source: Commun Chem. 2021 Nov 3;4:152. doi: 10.1038/s42004-021-00590-3 (PMC9814914; doi:10.1038/s42004-021-00590-3)
Supplement: Supplementary file 1 — Supplementary Information [file 42004_2021_590_MOESM1_ESM.pdf]

The interactive role of methane beyond a reactant in crude oil upgrading

Hao Xu, Zhaofei Li, Yimeng Li, Hua Song*

Green Catalysis Research Group, Department of Chemical and Petroleum Engineering, University of Calgary, 2500 University Drive, NW, Calgary, Alberta T2N 1N4, Canada

*Corresponding author

Fax: +1 (403) 284-4852; Tel: +1 (403) 220-3792; E-mail: sonh@ucalgary.ca

Supplementary Information

Contents

Fig. S1 N_2 adsorption isotherms of fresh and used catalysts with different reaction cycles under methane and nitrogen. 3

Fig. S2 NH_3-TPD patterns of fresh and used catalysts with different reaction cycles under methane and nitrogen. 4

Fig. S3 DTG patterns of used catalysts with different reaction cycles under methane and nitrogen. 5

Fig. S4 Optimized structures of nitrogen adsorption on the external surface and in the internal pore structure of the zeolitic catalysts through DFT calculation. Energy unit: kJ mol^{-1}. 6

Fig. S5 Optimized structures of *n*-butylbenzene (nBuB) adsorption on the external surface and in the internal pore structure of the zeolitic catalysts through DFT calculation. (a) ZSM-5 catalyst, (b) Mo/ZSM-5, (c) Ce/ZSM-5. Energy unit: kJ mol^{-1}. 7

Fig. S6 The typical profiles of (a) temperature and (b) pressure during the catalytic heavy oil upgrading process. 8

Fig. S7 The typical profiles of (a) temperature and (b) pressure during the catalytic *n*-butylbenzene conversion process. 9

Fig. S8 Other conformations of methane adsorption inside and outside the zeolitic pore structure. Energy unit: kJ mol^{-1} 10

Fig. S9 Other conformations of *n*-butylbenzene (nBuB) adsorption inside and outside the zeolitic pore structure. Energy unit: kJ mol^{-1}........ 11

Table S1 Properties of crude oil and liquid products after the upgrading process over MOU catalyst under methane and nitrogen........ 12

Table S2 Overall analysis results of *n*-butylbenzene reactions over catalysts with different reaction cycles........ 13

Table S3 Gas analysis of *n*-butylbenzene reactions over catalysts with different reaction cycles. 14

Table S4 Overall analysis results of *n*-butylbenzene reactions over regenerated catalysts. 15

Table S5 Gas analysis of *n*-butylbenzene reactions over regenerated catalysts. 16

Table S6 Calculation of the *n*-butylbenzene (nBuB) adsorption over ZSM-5 with and without dispersion corrections........ 17

Table S7 Additional physical and compositional properties of the crude oil. 18

Table S8 Contents of several typical metal impurities in crude oil and liquid products after the upgrading process. 19

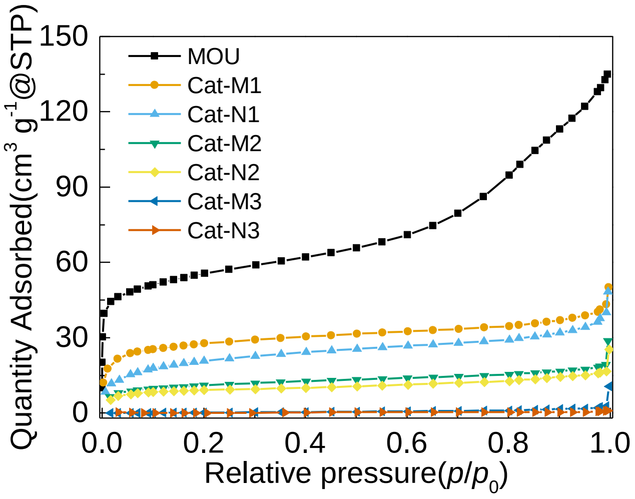

Fig. S1 N_2 adsorption isotherms of fresh and used catalysts with different reaction cycles under methane and nitrogen.

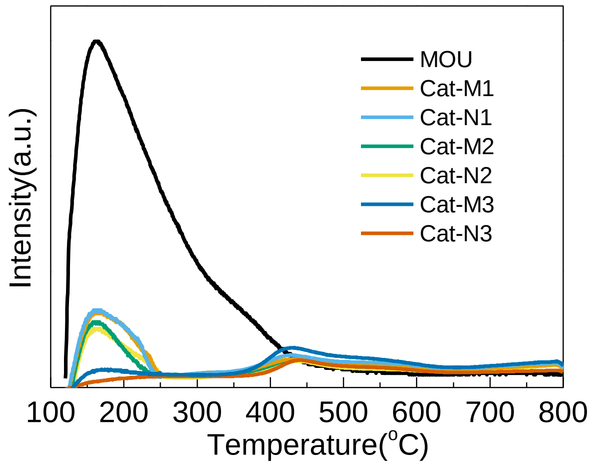

Fig. S2 NH_3-TPD patterns of fresh and used catalysts with different reaction cycles under methane and nitrogen.

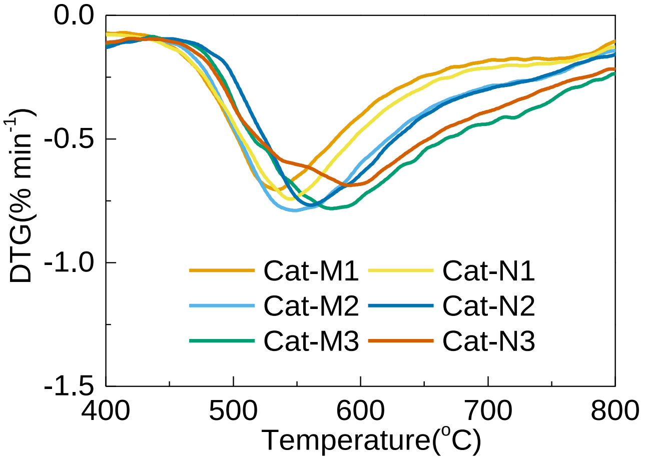

Fig. S3 DTG patterns of used catalysts with different reaction cycles under methane and nitrogen.

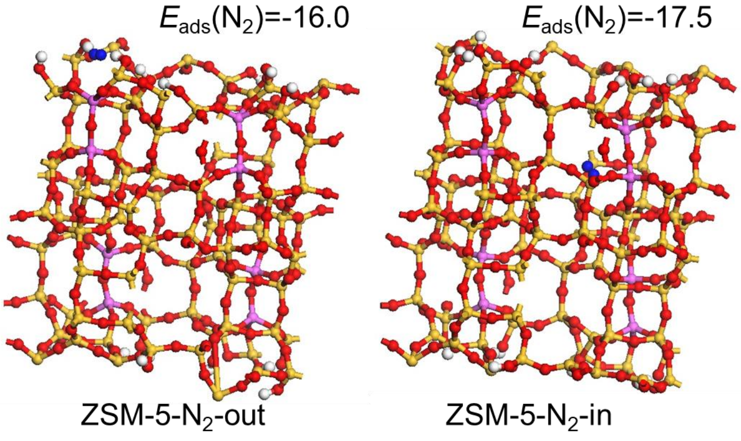

Fig. S4 Optimized structures of nitrogen adsorption on the external surface and in the internal pore structure of the zeolitic catalysts through DFT calculation. Energy unit: kJ mol^{-1}.

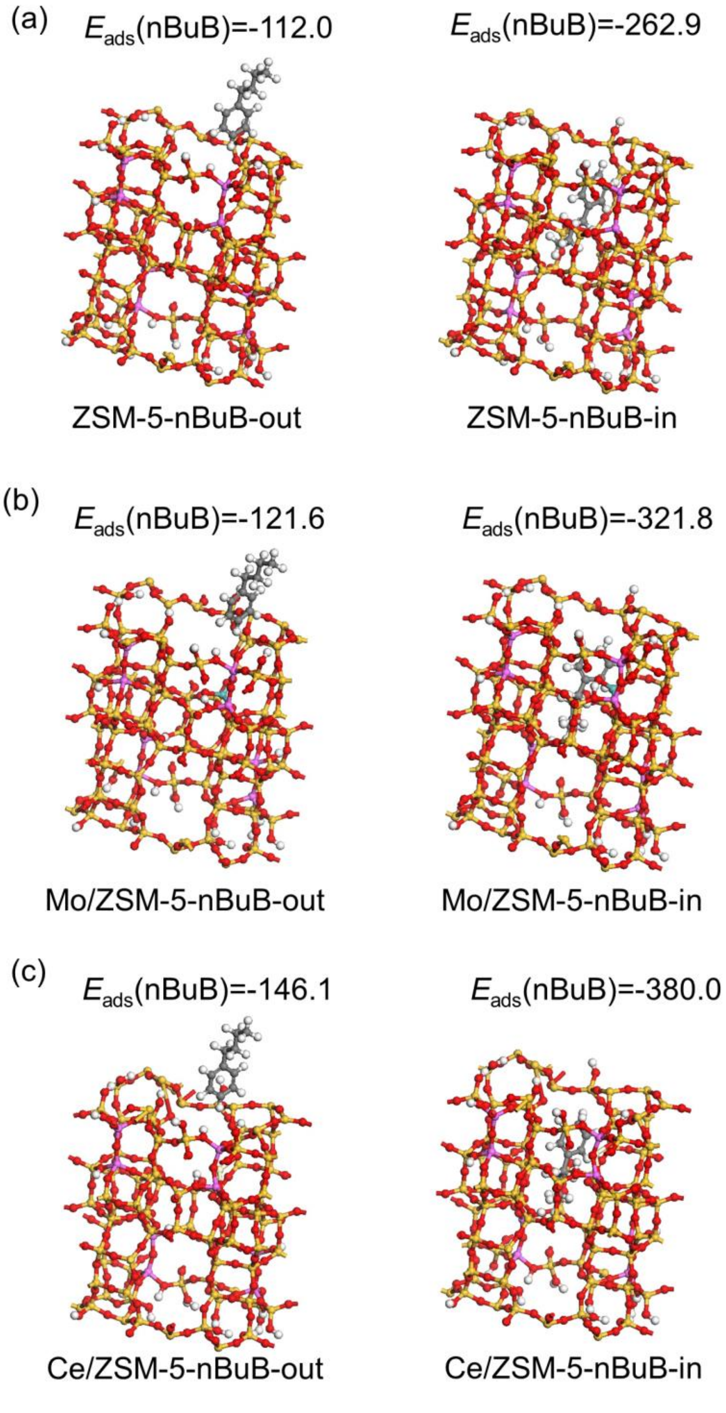

Fig. S5 Optimized structures of *n*-butylbenzene (nBuB) adsorption on the external surface and in the internal pore structure of the zeolitic catalysts through DFT calculation. (a) ZSM-5 catalyst, (b) Mo/ZSM-5, (c) Ce/ZSM-5. Energy unit: kJ mol^{-1}.

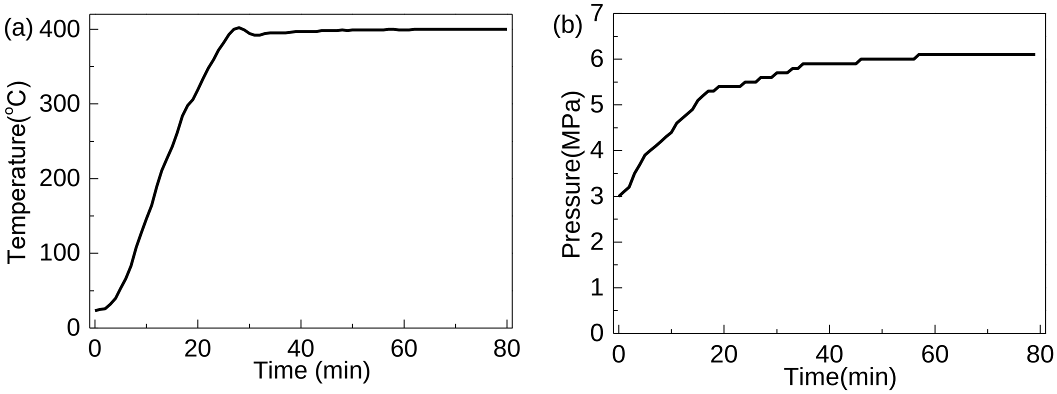

Fig. S6 The typical profiles of (a) temperature and (b) pressure during the catalytic heavy oil upgrading process.

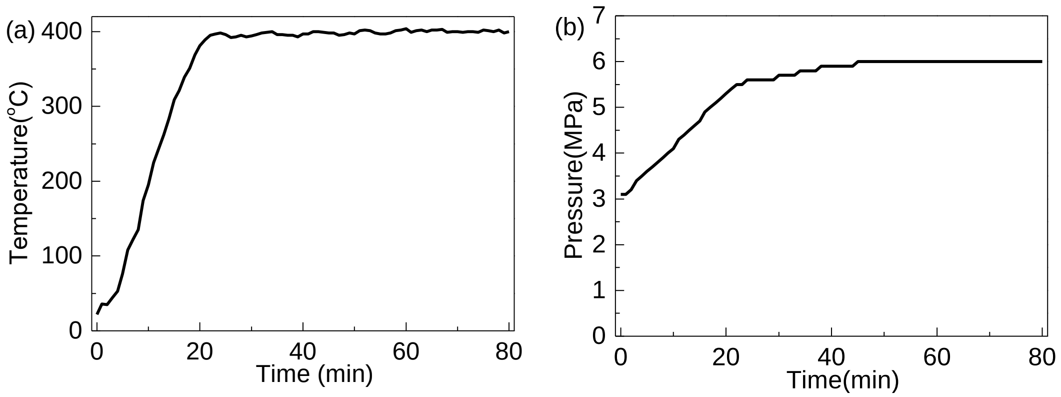

Fig. S7 The typical profiles of (a) temperature and (b) pressure during the catalytic *n*-butylbenzene conversion process.

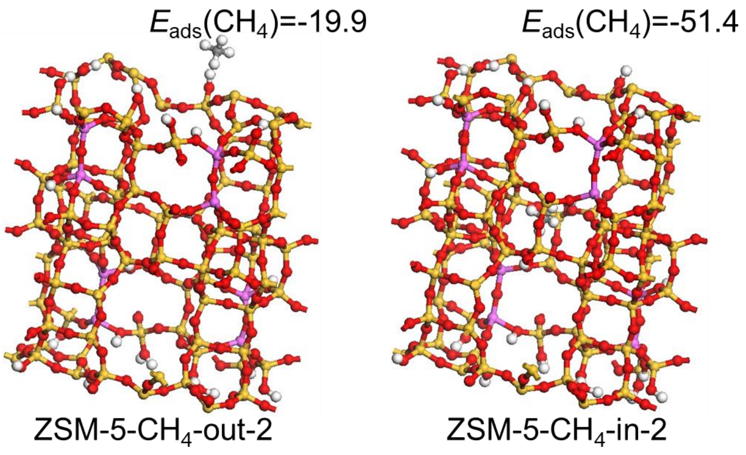

Fig. S8 Other conformations of methane adsorption inside and outside the zeolitic pore structure. Energy unit: kJ mol^{-1}

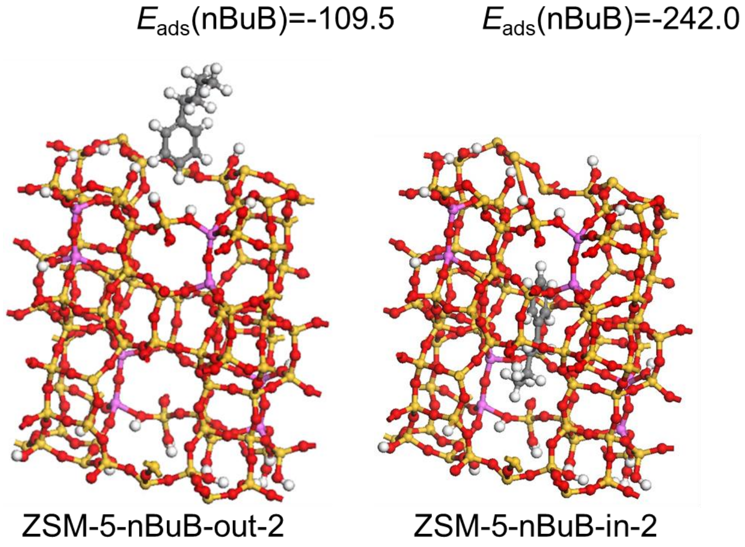

Fig. S9 Other conformations of *n*-butylbenzene (nBuB) adsorption inside and outside the zeolitic pore structure. Energy unit: kJ mol^{-1}

Table S1 Properties of crude oil and liquid products after the upgrading process over MOU catalyst under methane and nitrogen.

Property	Crude oil	CH_4			N_2		
		Prod-M1 Repeat 1	Prod-M1 Repeat 2	Prod-M1 Repeat 3	Prod-N1 Repeat 1	Prod-N1 Repeat 2	Prod-N1 Repeat 3
Overall mass balance (%)	/	99.5	99.9	99.9	99.9	99.7	99.1
Gas yield (wt%)	/	2.2	2.4	2.3	1.9	2.1	2.5
Liquid yield (wt%)	/	97.1	97.3	97.3	97.8	97.4	96.4
Coke yield (wt%)	/	0.23	0.23	0.24	0.24	0.22	0.22
Liquid viscosity (mPa s)	3373	105	102	105	151	147	150
Liquid density (g cm^{-3})	0.96570	0.94455	0.94523	0.94520	0.95167	0.95160	0.95170

Table S2 Overall analysis results of *n*-butylbenzene reactions over catalysts with different reaction cycles.

Entry	Butylbenzene conversion (%)	Gas yield (wt%)*	Liquid yield (wt%)	Coke yield (wt%)	Overall mass balance (%)
MOU	96±1	5±0.5	92±5	1.0±0.1	98±5
Cat-M1	50±1	2±0.2	97±5	0.8±0.1	100±5
Cat-N1	43±1	2±0.2	97±5	0.8±0.1	100±5
Cat-M2	41±1	2±0.2	95±5	0.7±0.1	98±5
Cat-N2	34±1	1±0.2	97±5	0.7±0.1	99±5
Cat-M3	36±1	1±0.2	95±5	0.6±0.1	97±5
Cat-N3	23±1	1±0.2	96±5	0.5±0.1	98±5
SiC	21±1	1±0.2	95±5	0.0±0.1	96±5

*All yields are based on *n*-butylbenzene feedstock.

Table S3 Gas analysis of *n*-butylbenzene reactions over catalysts with different reaction cycles.

Entry	CH_4 conversion (wt%)	H_2 yield (wt%)*	C_2 yield (wt%)	C_3 yield (wt%)	C_4 yield (wt%)
MOU	2.1±1.0	0.10±0.02	0.34±0.07	1.7±0.3	2.6±0.5
Cat-M1	1.3±1.0	0.05±0.01	0.80±0.16	0.20±0.04	0.78±0.11
Cat-N1	1.6±1.0	0.05±0.01	1.07±0.21	0.28±0.06	0.66±0.13
Cat-M2	1.4±1.0	0.04±0.01	0.90±0.18	0.12±0.02	0.72±0.14
Cat-N2	1.3±1.0	0.04±0.01	0.86±0.17	0.1±0.02	0.38±0.05
Cat-M3	1.1±1.0	0.03±0.01	0.96±0.20	0.08±0.02	0.31±0.06
Cat-N3	1.1±1.0	0.03±0.01	0.94±0.19	0.08±0.02	0.24±0.04
SiC	0.4±1.0	0.01±0.005	0.69±0.14	0.05±0.01	0.32±0.06

*All yields are based on *n*-butylbenzene feedstock.

Table S4 Overall analysis results of *n*-butylbenzene reactions over regenerated catalysts.

Entry	Butylbenzene conversion (%)	Gas yield (wt%)*	Liquid yield (wt%)	Coke yield (wt%)	Overall mass balance (%)
Cat-M1-R	93±1	4±5	95±5	0.9±0.1	100±5
Cat-N1-R	93±1	5±5	92±5	0.9±0.1	98±5

*All yields are based on *n*-butylbenzene feedstock.

Table S5 Gas analysis of *n*-butylbenzene reactions over regenerated catalysts.

Entry	CH_4 conversion (wt%)	H_2 yield (wt%)*	C_2 yield (wt%)	C_3 yield (wt%)	C_4 yield (wt%)
Cat-M1-R	1.9±1.0	0.06±0.01	0.17±0.03	1.6±0.3	2.3±0.3
Cat-N1-R	2.4±1.0	0.07±0.02	0.16±0.03	1.7±0.3	2.5±0.5

*All yields are based on *n*-butylbenzene feedstock.

Table S6 Calculation of the *n*-butylbenzene (nBuB) adsorption over ZSM-5 with and without dispersion corrections.

Dispersion Correction	ZSM-5 (a.u.)	nBuB (a.u.)	In/Out	ZSM-5-nBuB (a.u.)	Adsorption Energy (kJ mol^{-1})
No	-41680.239	-385.844	In	-42066.184	-262.9
No	-41680.239	-385.844	Out	-42066.126	-112.0
Yes	-41680.787	-385.910	In	-42066.804	-279.8
Yes	-41680.787	-385.910	Out	-42066.751	-141.9

Table S7 Additional physical and compositional properties of the crude oil.

Property	Crude oil
API gravity	15.0
Characterization K factor	9.3
Classification	Aromatic
TAN (mg KOH g^{-1})	1.06
Asphaltene content (wt%)	17.7
Olefin content (wt%)	BDL*
Carbon content (wt%)	85.07
Hydrogen content (wt%)	11.44
H:C ratio	1.61
Oxygen content (wt%)	0.57
Nitrogen content (wt%)	0.21
Sulfur content (wt%)	3.06
Ca (ppm)	49
V (ppm)	125
Ni (ppm)	63

*BDL: below detection limit

Table S8 Contents of several typical metal impurities in crude oil and liquid products after the upgrading process.

Oil sample	Ca (ppm)	V (ppm)	Ni (ppm)
Crude oil	49	125	63
Prod-M1	50	126	60
Prod-N1	50	128	61
Prod-M2	48	124	60
Prod-N2	50	128	58
Prod-M3	49	129	61
Prod-N3	51	130	60
